# “Recovering, not recovered” Hospital disaster resilience: a case-study from the 2015 earthquake in Nepal

**DOI:** 10.1080/16549716.2021.2013597

**Published:** 2022-02-09

**Authors:** Maria Moitinho de Almeida

**Affiliations:** Centre for Research on the Epidemiology of Disasters (Cred), Institute of Health and Society, Université catholique de Louvain, Brussels, Belgium

**Keywords:** Disaster management, disaster medicine, earthquake, hospital resilience, mixed-methods research

## Abstract

**Background:**

Disasters are an increasing threat to human health, but we know little about their impact on health services, particularly in low and middle-income settings. ‘Resilient hospitals’ have been increasingly recognized as a cornerstone of disaster management. While various frameworks of hospital resilience exist, they emerged from pre-disaster considerations, and do not incorporate evidence from post-disaster settings.

**Objective:**

This dissertation investigated the impact of a large-scale sudden onset disaster in a tertiary hospital in Nepal, and explored its resilience mechanisms.

**Methodology:**

This consists of an in-depth case-study combining quantitative data from routinely generated hospital records and qualitative data from semi-structured interviews with hospital staff. We used both advanced statistical methods and mixed inductive and deductive coding to analyze the data.

**Results:**

Most of the admitted earthquake victims required surgical interventions and long hospitalizations, considerably straining the hospital. For six weeks, the average number of daily admissions decreased. During this period, the share of injury-related admissions was particularly high, and such admissions were particularly long compared to the baseline. Admissions due to other conditions relatively decreased and were shorter. We found that the hospital’s resilience was highly dependent on emerging adaptations, in addition to the pre-existing disaster plan. Individual resilience of staff also played a major role, and was influenced by senses of safety, meaningfulness, and belonging.

**Conclusion:**

Hospitals should prepare resources and plan for their known disaster risks, but should also allow for a certain flexibility to innovative adaptions to emerging, unforeseen challenges. Challenges faced by hospital workers should not be undermined, and addressing them will increase hospital resilience.

## Background

Disasters are not natural. They consist of serious disruptions in the ‘functioning of a community or a society’ [1], and they result from interactions between a hazard, which can be natural, and the community’s vulnerability and coping capacity. Earthquakes are an example of large-scale, sudden-onset disasters, because they occur quickly and unexpectedly, and they can be so destructive that affected communities need external assistance [1]. Millions of earthquakes occur every year around the globe, but only small part of these are sufficiently strong to be measured. Of these, a very small fraction actually has human impact [2]. Between 2000 and 2019, 552 earthquakes with human impact were captured in the international disaster database (EM-DAT), having affected about 118million people [3]. Earthquakes are not distributed equally across the globe, and Asia is disproportionately affected, having hosted two thirds of earthquakes that occurred in the last 20 years [3].

Earthquakes carry important human health consequences. A major predictor of mortality is the built environment, and head and trunk injuries are typically deadlier than injuries to the limbs [4,5]. The number of injured victims can be very high after earthquakes, making hospital care an important aspect of disaster response. However, hospitals face several challenges and their ability to provide care can be seriously disrupted after large-scale sudden-onset disasters. They can suffer from building damage or even collapse, hospital staff can be affected or unable to reach the workplace, patients themselves may be unable to access the hospital, and there is a sudden increase of healthcare demand [6].

### Resilience of health services

It is hence important that hospitals are able to absorb the shock of disasters, retain their essential functions, surge their capacity to provide emergency care, and recover to their original or to a new adaptive state. In other words, resilient [7]. But resilience is a maturing concept and its definition remains elusive in multiple fields of knowledge [8].

With regard to health, resilience is often linked to a community’s or a system’s capacity to cope with and manage health risks while maintaining essential functions of health systems [9]. Health systems and health services are complex systems and can be influenced by external shocks that disrupt their functioning [10]. Health System Resilience can be presented in a ‘shock cycle’ that consists of four phases [8]:

1. System preparedness to shocks
2. Shock onset and alert
3. Shock impact and management
4. Recovery and learning

For a national or regional health system to be resilient, the health facilities that compose it must also be independently resilient, increasing the complexity of health system resilience. Different frameworks have separately attempted to define health system or health service resilience, and the fact that many have emerged in parallel further challenges operationalizing the concept of resilience. Table 1 [11] presents an overview of existing frameworks of hospital and health system resilience.

**Table 1.** Overview of major hospital and health system resilience frameworks [11]

Authors	Health system component	Domains of resilience
Bruneau et al, 2007 [14]	Hospital/ Acute healthcare facility	Means of Resilience:ResourcefulnessRedundancy Ends of Resilience:RapidityRobustness
Zhong et al, 2014 [7]	Hospital	Framework to evaluate hospital resilience:1. Hospital safety2. Command, communication, cooperation system3. Disaster plan4. Resource stockpile5. Staff capability6. Disaster training and drills7. Emergency services and surge capability8. Recovery and adaptation.
WHO, 2015 [9]	Health System	Context: the health system1. Challenge/disturbance: shock, stress2. Capacity to deal with disturbance3. Choices and opportunities4. Outcome options
Blanchet et al, 2017 [12]	Health System	Capacity to:1. Absorb2. Adapt3. Transform Derived from the system’s ability to manage:KnowledgeUncertaintiesInterdependenceLegitimacy
Kruk et al, 2017 [13]	Health System	Resilient Health Systems are:1. Aware: track threats, map weaknesses and strengths2. Diverse: address a broad range of health issues3. Integrated: cooperate with various sectors and involve the community4. Self-regulating: contain threats; stability5. Adaptive: respond to new challenges; rebound stronger than before

Hospital resilience was the first resilience concept to emerge in the domain of health systems and services. More comprehensive than ‘preparedness’ and ‘safety’, the concept of hospital resilience includes the actual disaster scenario. The engineering sciences were the first to study this phenomenon, and used the 4 R framework to conceptualize hospital resilience [14–16]. This framework consists of ends of resilience (Robustness and Rapidity) and means of resilience (Redundancy and Resourcefulness). Robustness is the strength to withstand a given level of stress without suffering degradation or loss of function. Rapidity means that priorities are met

and goals are achieved in a timely manner, in order to contain losses, recover

functionality, and avoid further disruption. Redundancy is the ability to replace disrupted elements, and Resourcefulness is the capacity to mobilize and use resources, including through coordination [14–16].

But these concepts needed to be easily interpreted by hospital managers and health decision-makers, which led to the research conducted by Zhong and colleagues [7,17,18]. They found that a hospital’s resilience depended on structural and non-structural components, as well as emergency medical functions and disaster response capacity [7] and, they identified four primary domains where the 4 R dimensions could be applied: (i) hospital safety and vulnerability, (ii) disaster preparedness and resources, (iii) continuity of essential services, and (iv) recovery and adaptation. The empirical work that followed to develop a hospital resilience assessment tool included expert consultations [18] and a pilot test in 41 tertiary hospitals in China [17]. A more recent systematic review identified a set of hospital resilience indicators that could also be linked to the 4 R dimensions [19].

However, while research is ongoing and advancing this unique field of knowledge, many questions remain. First, we know little about how disasters affect the functioning of hospitals, particularly in low and middle-income settings. Second, while hospital resilience frameworks exist, they were not developed from actual disaster contexts. This means that we don’t know whether they actually picture what happens after disasters occur. It is a critical limitation as resilience is more comprehensive than just preparedness, and includes phases such as response and recovery. These important research gaps hinder optimal disaster management, and addressing them can substantially reduce the human impact of disasters.

### Aim and objectives

This dissertation investigated the impact of an earthquake on the functioning of a tertiary hospital in Nepal, and explored hospital resilience mechanisms. The specific objectives were:

1. To study the clinical and demographic profile of earthquake victims who were admitted in the hospital, and what influenced their length of hospital stay (LOS)
2. To compare hospital admissions before and after the earthquake, and estimate the effect of the earthquake on admissions
3. To assess the impact of the earthquake on the hospital functioning, and explore resilience as experienced by hospital staff.

## Methods

### Design

This dissertation consisted of an in-depth case-study that combined quantitative and qualitative methodologies, allowing to explore a complex phenomenon in its real-life context [20]. It is based on a series of three articles, each focusing on a different study addressing a specific objective. Table 2 presents an overview of the studies, their methodologies, and their relation to the health system resilience shock cycle. Study I and II both used complementary datasets containing information about patient admissions in the hospital. Study III collected qualitative information through 18 in-depth interviews with hospital staff from different professions and seniority levels; 7 of those interviews were conducted in Nepali with an interpreter present, and the transcripts were later translated into English. For this study, we used a mixed deductive and inductive approach, using the 4 R resilience framework [14–16], which was theoretical framework of this thesis.

**Table 2.** Overview of studies composing the dissertation presented in this article

	Study I [32]	Study II [34]	Study III [39]
Objective	Objective I: To study the profile of admitted earthquake victims, and what influenced Length of hospital Stay	Objective II: To compare hospital admissions before and after the earthquake, in terms of:Length of hospital stayDiagnostic categoriesTotal admissions	Objective III: To assess the impact of the earthquake on the hospital functioning, and explore resilience as experienced by hospital staff
Corresponding health system resilience shock cycle phase	Shock impact	Shock impact	1) System preparedness to shocks 2)Shock onset and alert 3)Shock impact and management 4) Recovery and learning
Data source	Centralized datasets and patient registries	Centralized datasets and patient registries	In-depth interviews with hospital staff
Main analysis method	Time-to-event analysis of length of hospital stay	Negative binomial regressionsLogistic regressionsGeneralized Additive Models	Mixed coding:Deductive for hospital system resilience (4 R)Inductive for burden and individual resilience
Sample size	n = 501	n = 9,596	n = 18 (purposive sample)

The complementary use of different sources and type of data is believed to increase the internal validity of a case-study, and helps create a holistic picture of the topic under study. Using mixed-methodology in health service research and humanitarian contexts also allows to gain a comprehensive view of complex phenomena [21]. Understanding contextual factors is also critical in case-studies, and this is more successful with field work and associated immersion, observations and interactions [22]. In this case, field work was an essential aspect to develop the studies presented in Table 2.

### Setting

The focus of this work was a reference tertiary hospital in Nepal, a lower middle-income country landlocked between India and China that faced political instability for decades until 2008. Nepal is considered at high risk for humanitarian crises and disasters [23], and the entire territory is regularly affected by disasters with reported human impact [3]. While maternal and child mortality indicators have seen steady improvements over the years [24], several health challenges persist in Nepal. For instance, hospital care is only free for people in verified poverty situations and other vulnerable groups [25,26], leaving a substantial share of the population paying out-of-pocket for health care. On Saturday, 25 April 2015, a high-magnitude earthquake Nepal, followed by many aftershocks, a major one on May 12^th^. This series of earthquakes killed nearly 9,000 people and injured another 22,000 [27]. Almost one third of the country’s population was affected by the disasters, and about 84% of the health services in the affected districts were destroyed or damaged [28]. The study hospital, located in the capital city Kathmandu, was built with earthquake-resistant standards and had a disaster plan in place [29,30], having played a major role in the earthquake response [31].

## Results

### Study I: what is the profile of earthquake victims who needed hospital admission?

We studied the profile of 501 earthquake victims who were admitted in the study hospital [32]; 254 (51.2%) were women and 17.2% (n = 85) were children aged 0–14 years. Nearly half (n = 195, 48.9%) had a lower limb injury as main diagnosis of admission, and two thirds (n = 226, 65.7%) needed orthopedic surgery. Fractures represented 65.8% of all injury cases (n = 288). The most common cause of admission were femur and lower leg fractures, accounting for 26% of all earthquake victim admissions. For diagnoses not belonging to the injury group, the most common cause of admission was coded as ‘post-surgical states’. Date of admission ranged between 0 and 166 days after the first earthquake of April 25^th^; the peak occurred five days after the earthquake with 77 admissions. In 37 cases (7%), death was reported as an outcome.

The median length of stay in the hospital was 10 days, and the mean was 14.7 days. We first conducted bivariate log-rank tests, and found that demographic variables were not associated with length of hospital stay. We calculated individual hazard ratios for the variables that showed a significant association with length of stay, and they are presented in Table 3. Longer hospitalizations were associated with lower limb and trunk injuries, crushing injuries, and undergoing an amputation or plastic surgery.

**Table 3.** Measures of association (unadjusted hazard ratios) of different characteristics with hospital length of stay

Variable	*HR (95%CI)*	*p*
Body Region		
Head and Neck	Ref	
Lower limb	0.68 (0.51–0.91)	**0.009**
Trunk	0.62 (0.44–0.87)	**0.005**
Upper Limb	0.99 (0.68–1.16)	0.991
Crushing		
No	Ref	
Yes	0.57 (0.36–0.89)	**0.013**
Amputation		
No	Ref	
Yes	0.65 (0.43–0.99)	**0.045**
Surgery Type		
Orthopaedics	Ref	
Neurosurgery	0.90 (0.64–1.26)	0.542
Plastic surgery	0.57 (0.38–0.85)	**0.006**
Wound care	1.42 (0.87–2.34)	0.163
Other	0.84 (0.56–1.25)	0.382

HR: hazard ratio (unadjusted); 95%CI: 95% confidence interval; Z: Z-score; p: p-value; Ref: reference category. Significant p-values (lower than 0.05) are presented in bold. This table is adapted from [32].

Consistent with the literature, the majority of the victims who made it to the hospital and were hospitalized had orthopedic injuries and underwent surgical intervention. This is probably because such injuries are more survivable, as opposed to injuries to the head, chest or abdomen [5]. However, in this study earthquake victims have particularly long hospitalizations; information from the other two studies provides additional insights into this finding. Another finding that merits attention is the fact that children are underrepresented in this sample in comparison to the population distribution in Nepal at the time of the earthquake, which was estimated at 33.4% [24]. This can be because the earthquake occurred on a Saturday during the day, and children were not in school neither sleeping, so they were not as affected as they could have been. But another plausible explanation is the fact that children have growing bones which are more resistant than adult bones, and when they sustain a fracture they don’t need surgery as often as adults do [33]. This would mean that many fractures in children did not warrant inpatient treatment, and are hence not reflected in admission data.

### Study II: how were hospital admissions affected with the earthquake?

We included 9,596 admissions occurring between March 15^th^ and 17 August 2015, and defined four periods of analysis: a pre-earthquake baseline (pre-EQ), acute (EQ1), post-acute (EQ2), and post-earthquake period (post-EQ). EQ1 and EQ2 were three-week intervals after the April 25^th^ earthquake. The rationale for this approach is explained elsewhere [34].

Overall, the most common causes of admission were injuries, pregnancy-related conditions, diseases of the digestive system, respiratory diseases, genitourinary diseases, and factors influencing health status and contact with health services. The post-EQ period contained 49% of all admissions, followed by the pre-EQ period with 26%. Women accounted for 56% of all admissions, while children under 15 years of age represented 17% of all admissions.

Average length of stay (LOS) was significantly longer in EQ1 than during pre-EQ (9.80 vs. 7.05, respectively, p < 0.001). This was particularly the case for injury-related admissions, where LOS increased by 57.3% (CI: 37.0–80.7; p < 0.001), whereas LOS for respiratory diseases was 21.6% shorter in EQ1 (CI: 7.1–34.6; p = 0.008).

In EQ1, the odds of injury admissions increased (aOR = 5.33, CI: 4.44–6.40), while they decreased for the majority of other diagnoses. Pregnancy-related admissions relatively decreased in EQ1 and remained low until post-EQ. The total number of admissions dropped in EQ1 and EQ2, and returned to pre-EQ trends in post-EQ. We estimate that there were in total 381 fewer admissions in this six-week period (CI: 206–556).

These results consolidate the findings from the previous study. The injury patterns seen after the earthquake in our study hospital required particularly long hospitalizations compared to before the earthquake. This may be related to injury characteristics, associated with high-energy trauma, and to the fact that this is a reference hospital and this is probable a selected sample of more severe cases. Two other findings merit attention: the relative but sustained decrease of respiratory and pregnancy-related conditions. In fact, previous work has reported an increase of respiratory diseases after earthquakes [35,36]. One explanation is the fact that respiratory conditions sustained would not require hospitalization, and would rather be reflected in outpatient care. Indeed, a study in a hospital near Kathmandu found an increase in emergency department visits due to respiratory diseases [37]. Such an explanation is not plausible for pregnancy-related admissions, and it may indicate that pregnant women are not receiving skilled care and deliveries are not conducted in health facilities. We elaborate on this finding in light of the qualitative results and the broader literature in the general discussion section.

### Study III: how did staff experience hospital resilience?

Following recommendations for health service research [38], we used a mixed deductive and inductive approach to analyze the data from the 18 interviews, with the starting themes from the 4 R resilience framework. The context of the interviews and characteristics of the interviewees are detailed elsewhere [39]. We categorized the burden to the hospital into material challenges, challenges to health service provision, challenges to management and coordination, and emotional and physical impact on individuals. Material challenges included shortages of medicines and of surgical and rehabilitation equipment. The high influx of injured victims created challenges to health service provision, as the capacity to treat trauma conditions was overwhelmed. Challenges to management and coordination occurred for a variety of reasons, but one aspect is that the earthquake occurred on a Saturday, senior staff were absent, and junior staff who were present were hence less likely to know the disaster plan. Individual staff experienced an increased workload in difficult conditions, while they were also concerned with their personal and family situations.

#### Ends of resilience

In terms of robustness, the hospital maximized capacity to provide emergency care, interrupting routine or elective activities. But questions regarding maintenance of quality of care arose, as well as concerns that patients were discouraged to travel for deliveries and other essential care.

During that time, we were not focusing on quality of care. (…) We had a lot of wound infections, we were not taking care of sterility properly … We just needed to provide care, we were focusing on life-saving and limb-saving activities.

We identified three stages of hospital rapidity. Critical rapidity was the time needed for the hospital to start essential work and assist injured victims while also self-organizing.

We tried to manage the pharmacy without a software system, but for two days, we failed. We were almost out of stock after two days. Then we started to ask for medicine supply from different agencies, from the government …

After this reorganization, stabilizing rapidity allowed the hospital to address earthquake-related surges in a new, stable rhythm, until routine activities restarted and the hospital reobtained a ‘normal look’.

(…) That made us feel like “ok we are back into function”: no patients treated on the ground, all patients treated in the wards.

After routine activities restarted, time was still needed to recover to a new, non-emergency phase and feeling. We found that recovery rapidity was subjective and person-specific, with many interviewees struggling to explain their experiences of ‘recovery’ – some even mentioned they were *still recovering, and not recovered*.

#### Means of Resilience

The hospital found suitable alternatives to many disrupted elements. An example of its redundancy is that it established linkages with ‘step-down centers’ to refer patients no longer requiring advanced hospital care, which liberated beds to accommodate severe cases.

Looking at resourcefulness, the pre-existing disaster plan and trainings were important, but many if not the majority of adaptations were spontaneous, compensating for a perceived lack of coordination. Many new partnerships or collaborations were established with external organizations, health services were rearranged, and staff changed their tasks or assumed new roles to adapt to emerging situations.

*At a disaster time, everyone needs to know their job. But I did not know my job: the scenario drove me to that job.*

#### Individual resilience

During our analysis, it became evident that hospital staff were essential to the resilience of the hospital as a whole. But the resilience of staff as individuals could not be analyzed in light of the 4 R framework, which is designed for systems. We identified three major determinants of hospital staff resilience: safety, meaningfulness, and sense of belonging. Feeling safe allowed staff to continue working despite recurrent aftershocks, and seemed to influence full recovery. Meaningfulness helped making sense of the tireless work, the putting family second, the constant fear. Interviewees who did not feel their experiences were meaningful were more often frustrated, or felt trapped in their work. In general, interviewees felt that family cohesiveness in Nepal was an important aspect, allowing them to leave their loved ones with extended families or with friends or neighbours. This contributed to cultivating a sense of belonging to a supportive community.

We were terrified, but we knew that we were safe in ICU because that building was safe.

After the second day I shifted my family to uncle’s house (…). They had like a family get-together. And I was free to work.

## Discussion

### Earthquake impact

In our study, earthquake victim admissions were particularly long (mean = 14.7 days; median = 10) compared to studies in other hospitals, either in Nepal [40] (median = 8 days) or in China after the 2008 earthquake [41] (mean = 7 days). A study in Italy after the L’Aquila earthquake in 2009 found that the average length of hospital stay (LOS) of admitted earthquake victims was 12.11 days; LOS was significantly associated with age, in a sample where 57% of patients were older than 60 years [42]. In our sample, only 29% of patients were older than 50 years, and age was not associated with length of stay, suggesting a different cause for the long hospitalizations. Study II showed that in the three weeks after the earthquake, injury admissions were significantly longer than before the earthquake. This long LOS is probably related to the fact that we focused on a reference hospital that received more severe cases, and that people from remote districts reached the hospital with considerable delay, probably with more advanced conditions. Previous research on earthquake injuries found that length of hospital stay was associated with the level of resource use [41], suggesting that our study hospital was particularly strained during the earthquake response.

Was the hospital overwhelmed? Our studies show some nuances. The total number of admissions decreased in the six weeks after the earthquake. Pressure points were elsewhere, not reflected in hospital admissions. Reports show that a total of 1,723 injured victims were treated at TUTH [43], but less than a third were admitted. The cases that needed admission presented a specific profile: from a share of 11.1% of all admissions in the pre-earthquake period, injuries represented 38.5% of all admissions in the 3 weeks after the earthquake (aOR = 5.3, p < 0.001). The average length of stay also significantly increased during the same period, and mostly due to injury-related admissions, which were significantly longer. The majority of admitted earthquake victims required a surgical intervention (69%, n = 345), with many needing reinterventions. Staff reported that operation theatres were constantly occupied with earthquake-related surgeries, and new intensive care beds had to be set up. To deal with this sudden increase of demand for surgical care, non-urgent activities were put on hold. As explained in a conceptual model by von Schreeb et al., the need for hospital care due to injuries is concentrated in the days after a sudden-onset disaster, while other elective and less urgent conditions are deferred [44]. We lack information on which exact activities were cancelled or postponed, and we are hence unaware of which were time-sensitive conditions, like cancer surgical care. We can only assume that this was the case for all non-urgent care, which would then substantially aggravate the negative consequences of the earthquake. Compensating for this interrupted care I quite a complex endeavor. A study from the COVID-19 pandemic modeled that that clearing the backlog of elective surgical care after the first lockdown could last up to 45 weeks if the surgical volume increased by 20% [45].

A concerning finding from study II is the sustained decrease of pregnancy-related admissions. This was confirmed in Study III by perceptions of hospital staff, who believed this reduction was due to insufficient delivery beds and preference of pregnant women to use services closer to their residence. One study shows, after the earthquake, women in rural Nepal preferred to deliver at home rather than at a health facility, seriously challenging referral in case of complications [46]. This finding is not unique to the Nepal earthquake context, and there is evidence of reduced pregnancy-related admissions after disasters in other settings [47], or of worse maternal outcomes in general [48,49]. Possible explanations in the literature include reduced access to hospitals and health facilities [47], the death of many skilled attendants [49], and the lack of specific provisions for women and children in disaster plans [48].

### Hospital function and resilience

Studies I and II attempted to measure the burden to the hospital and the changes in function, and were complemented by the qualitative information obtained in Study III. These findings can be put in perspective with the conceptual models proposed by von Schreeb et al. (2008) and Zhong et al. (2014) [7,44], and may contribute to future studies that attempt to measure hospital resilience, or the lack of it. Study III is one of the first to use the well-established 4 R system resilience framework as a starting point to explore mechanisms of hospital resilience in a post-disaster setting, as experienced by its staff. We captured a richness of experiences and complexity of events that the 4 R framework failed to reflect. For instance, the importance of emerging adaptations even when a disaster plan exists is not really featured in this framework. This can be a consequence of the fact that the majority of the literature, both empirical and theoretical, is actually generated from pre-disaster contexts. Although recent work highlights the need of ‘adaptive flexibility’ [17] or ‘adaptive capacity’ [50], these concepts remain vaguely defined in the scientific literature. Moreover, while previous studies have demonstrated the important role of staff experiences in hospital disaster response [51,52], ours is the first to identify individual resilience of hospital staff in the frontlines as an important contributor to hospital resilience. In our study, we identified three major determinants of hospital staff resilience: meaningfulness, sense of safety, and sense of belonging. The importance of staff feeling safe in hospital disaster response had already been identified in a study after Typhoon Haiyan [51]. In line with the literature, making sense of a difficult experience is important for hospital staff to believe efforts were worthwhile; and the safety recurrently mentioned by health and humanitarian workers in times of emergencies [53,54,^54^].

### Global implications

Several recommendations can be made for global disaster and hospital management practices. While important, structural resistance alone is not sufficient to ensure resilience of health services; functional aspects, if not well managed, can create major bottlenecks.

Earthquake-prone countries should have strategies that ensure sufficient equipment to treat high numbers of orthopaedic injuries, and that strengthen surgical capacity in peripheral services. To achieve this, diplomatic agreements with neighbouring countries may be required in order to improve efficiency. If able, tertiary hospitals should provide advanced care to disaster victims, but disruption of non-earthquake specialized care should be minimized.

After any type of disaster, the population and frontline workers experience great levels of suffering and stress that can seriously impact their mental health and ability to reach their full potential in the future [55]. Hospital disaster plans should have specific provisions to ensure appropriate and skilled support to their own staff. Strategies that contribute to staff wellbeing in times of disaster response also increase hospital resilience. This could be leveraged in international initiatives such as the ‘Hospitals Safe from Disasters’ Campaign [56] or the HOPE network [29].

Finally, the perspectives from different stakeholders should be fed into hospital disaster plans when they are being designed. This can greatly increase adherence to such plans and their effectiveness.

### Relevance for the COVID-19 pandemic

As the results of this work were being finalized, the world was hit by a pandemic disease caused by a novel coronavirus. As of early March 2021, more than 116 million people have been infected with this virus, and nearly 2.6 million have died from the novel coronavirus disease (COVID-19) [57]. Hospital and health system resilience became extremely relevant as we witnessed the collapse of critical care facilities and the prolonged interruption of non-emergency care, in rich and poor settings. It became evident that health facilities cannot face crises without engaging and collaborating with other actors in health systems, highlighting the complexity of health system and health service resilience. The pandemic also emphasized that crisis response plans could be obsolete if there is no adequate follow-up, and institutions must adapt as the situation evolves.

Societies also became more aware of the important role of frontline workers such as hospital staff during crisis response. As shown in our study, their resilience is critical for the resilience of a health service and a society as a whole. In the first wave occurring in early 2020, adequate personal protective equipment was lacking, putting staff at great risk of acquiring a severe infection, and transmitting it to their household members. This is a serious challenge to staff safety [54], and influences their ability to work, even if unconsciously. But during this period, countless global movements of solidarity were occurring, celebrating the courage of healthcare workers and valuing their work, ultimately contributing to a sense of belonging. However, as the situation is getting more and more protracted with recurrent waves of infection, ‘pandemic fatigue’, or ignoring preventive measures, is a dangerous threat to resilience of healthcare workers. The lack of collaboration from the general public can make staff feel their efforts are in vain and that the community is overlooking their needs.

## Conclusions

Our findings empirically support conceptual models of disaster impact on hospital care and show concrete, measurable changes in hospital function. This is the first research work to further explore the suitability of the 4 R framework on hospital disaster resilience through empirical post-disaster data collection and analysis. We argue that resilience is only evident after a disaster occurs, when unplanned adaptations emerge and individual staff face unique challenges. We recommend additional case-studies to quantify the short and long-term impacts of different disaster types on hospitals in different contexts, and to identify the main concepts that can be measured and used to predict resilience before a disaster happens. By producing evidence from different events and contexts, we will be able to differentiate contextual factors that influence resilience from other factors that are more easily modifiable. These could be further elaborated through the co-construction of a hospital resilience assessment tool where diverse stakeholders are engaged.

## Paper context

Resilient hospitals are essential to reduce the health consequences of disasters, but few studies examine the disaster impact on hospitals, and research on hospital resilience is mostly from pre-disaster conceptualizations. This article shows the complex and nuanced impact of a high-magnitude earthquake on a tertiary hospital in Nepal, and documents the resilience of the hospital as experienced in the frontlines.
